# Neuroprotection by the Ketogenic Diet: Evidence and Controversies

**DOI:** 10.3389/fnut.2021.782657

**Published:** 2021-11-23

**Authors:** Sarah M. Gough, Alicia Casella, Kristen Jasmin Ortega, Abigail S. Hackam

**Affiliations:** Bascom Palmer Eye Institute, University of Miami Miller School of Medicine, Miami, FL, United States

**Keywords:** neurodegeneration, ketogenic diet, mitochondria, ketone bodies, inflammation

## Abstract

The ketogenic diet (KD) is a high-fat low-carbohydrate diet that has been used for decades as a non-pharmacologic approach to treat metabolic disorders and refractory pediatric epilepsy. In recent years, enthusiasm for the KD has increased in the scientific community due to evidence that the diet reduces pathology and improves various outcome measures in animal models of neurodegenerative disorders, including multiple sclerosis, stroke, glaucoma, spinal cord injury, retinal degenerations, Parkinson's disease and Alzheimer's disease. Clinical trials also suggest that the KD improved quality of life in patients with multiple sclerosis and Alzheimer's disease. Furthermore, the major ketone bodies BHB and ACA have potential neuroprotective properties and are now known to have direct effects on specific inflammatory proteins, transcription factors, reactive oxygen species, mitochondria, epigenetic modifications and the composition of the gut microbiome. Neuroprotective benefits of the KD are likely due to a combination of these cellular processes and other potential mechanisms that are yet to be confirmed experimentally. This review provides a comprehensive summary of current evidence for the effectiveness of the KD in humans and preclinical models of various neurological disorders, describes molecular mechanisms that may contribute to its beneficial effects, and highlights key controversies and current gaps in knowledge.

## Introduction

The ketogenic diet (KD) and related diets have been used for many decades for weight loss, managing metabolic disorders and reducing seizures in pediatric epilepsy. Recent convincing evidence for neuroprotective effects of the KD in animal models of neurologic diseases has led to a surge in interest in testing the benefits and mechanisms of action of the diet. PubMed articles using the search term “ketogenic diet” increased nearly three-fold from 2010 to 2021 compared to all years prior to 2010, indicating heightened attention to the KD within the scientific community. The KD is used in clinical practice for several non-neurological conditions, including heart disease, diabetes, obesity, autism, glioblastoma and other cancers. However, other than for epilepsy, the KD is not yet recommended for reducing symptoms and slowing degeneration in any neurological disease. The objective of this literature review is to provide a comprehensive summary of supporting evidence from clinical and experimental laboratory studies on the effectiveness of the KD in neurodegenerative disorders of the brain, spinal cord and retina. Furthermore, we describe the current understanding of molecular mechanisms that may directly contribute to the beneficial effects of the KD and highlight key knowledge gaps.

## Ketone Body Metabolism: Ketogenesis and Ketolysis

The classic ratio of fat to carbohydrate plus protein in the KD is 3:1 or 4:1, in which 80–90% of total calories comes from fats, 4% from carbohydrates and 6% from proteins. Modifications of the diet to increase palatability and improve compliance are common, although may complicate drawing comparisons among different studies. These modifications include varying the relative amounts of macronutrients, the nature and sources of the fats and duration of the diet, while maintaining the appropriate fat to carbohydrate ratio. The general goal of classic and modified KDs is to achieve ketosis, a state in which levels of ketone bodies are elevated in the blood. Ketone bodies are chemically related water-soluble molecules that are generated by normal physiological metabolism of fatty acids by β-oxidation. The most well-known ketone bodies are beta-hydroxybutyrate (BHB), acetoacetate (ACA) and acetone. Typical circulating levels of ketone bodies within the blood are 100–250 μM, whereas physiological or nutritional ketosis leads to elevated ketone body levels in the range of 0.5–5 mM. In contrast, blood levels of ketone bodies in pathological ketoacidosis can reach up to 15–25 mM. Levels of blood and urine ketones are often measured to assess adherence to the diet, although ketone concentrations do not always correlate with better outcomes (1, 2).

**Figure 1:**
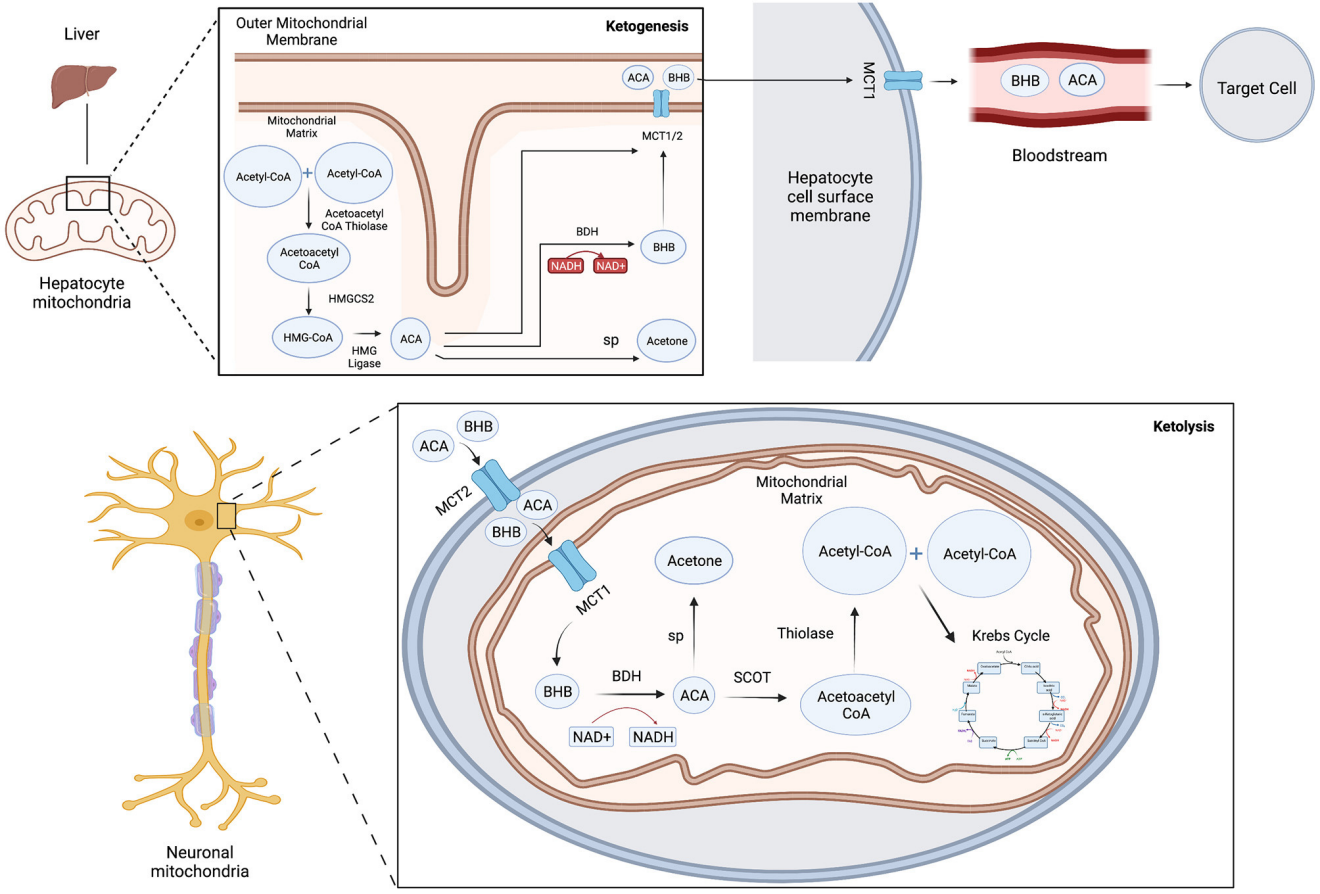
Schematic model of ketone body metabolism. Ketogenesis: Acetoacetyl-CoA thiolase converts two acetyl-CoA molecules into acetoacetyl-CoA, which is combined with another acetyl-coA then converted into beta-hydroxy-methylglutaryl-CoA (HMG-CoA) by HMG-CoA synthase 2 (HMGCS2), which is metabolized into ACA and acetyl-CoA by HMG-CoA lyase. ACA is converted into BHB by β-hydroxybutyrate dehydrogenase (BDH) or converted into acetone by spontaneous decarboxylation (sp). ACA and BHB are transported by monocarboxylate transporters (MCT) out of the liver into the blood. Ketolysis: ACA and BHB enter cells through MCT transporters and are reconverted with the mitochondria into acetyl-CoA and utilized in the Krebs cycle to yield GTP and ATP. BHB can also be converted back into acetoacetate by BDH, which generates NADH to help power the electron transport chain.

Ketone bodies are produced in a process known as ketogenesis, which generates ACA and BHB for use as alternative metabolic fuel sources for the body when glucose stores become depleted. Ketogenesis occurs during glycolytic inhibition from periods of fasting, intense exercise or severe carbohydrate restriction such as in the KD. Ketogenesis primarily occurs in the mitochondria of liver hepatocytes (3), in several neuronal cell types and in the retinal pigment epithelium (4), although the relative importance of extrahepatic ketogenesis is not fully known. Fatty acids derived from the diet are converted *via* β-oxidation reactions into acetyl-CoA, which enters the TCA cycle or is converted further into ketone bodies by several modulating enzymes and co-factors (Figure 1) (3). Acetoacetyl-CoA thiolase converts two acetyl-CoA molecules into acetoacetyl-CoA, which is combined with another acetyl-coA and is then converted into the intermediate product beta-hydroxy-methylglutaryl-CoA (HMG-CoA) by the key rate-limiting enzyme HMG-CoA synthase 2 (HMGCS2). HMG-CoA is metabolized into ACA and acetyl-CoA by the enzyme HMG-CoA lyase. Finally, ACA is reversibly converted into BHB by β-hydroxybutyrate dehydrogenase coupled with NADH oxidation (5) or converted into acetone by spontaneous decarboxylation. ACA and BHB are transported by monocarboxylate transporters (MCT) out of the liver into the blood where they are taken up and used by the brain and other organs through ketolysis.

Ketolysis is the metabolism of ketone bodies ACA and BHB for energy within the mitochondria. Ketone bodies enter extrahepatic cells down a concentration gradient through MCT transporters, are reconverted into acetyl-CoA and utilized in the citric acid cycle to yield GTP and ATP. BHB can be converted back into acetoacetate by BHB dehydrogenase coupled with the reduction of NAD+. This yields NADH to help power the electron transport chain. Additionally, although primarily exhaled, acetone may be metabolized into usable energy sources such as pyruvate, lactate and acetate.

## Evidence for Beneficial Effects of KD in Neurological Diseases

Many claims have been made about the benefits of the KD for overall health. However, the only well-established use of the KD for managing a neurological disease is for seizure reduction in pediatric epilepsy (6). Intriguingly, evidence for neuroprotection in other neurologic diseases from the classic and modified KD has been reported in pilot clinical studies and in pre-clinical laboratory models, as described below and summarized in Tables 1, 2. Note that studies using ketogenic-like diets that deviated from high fat/low protein/low carbohydrate content ratio, such as diets with excess fat but with normal carbohydrate levels, are not included in the summary below.

**Table 1:** Beneficial effects of the KD or ketone bodies in animal models.

**Disease**	**Animal model**	**Treatment**	**Outcome**	**References**
Alzheimer's disease	3xTgAD transgenic mouse	Diet supplemented with BHB	Reduced oxidized proteins and lipids	(7)
	APP/PS1 and Tg4510 transgenic mice	KD for 3 months	Improved locomotor activity, no improvement in learning or change in amyloid or tau deposition	(8)
	3xTgAD transgenic mouse	Ketosis induced by 2-deoxy-D-glucose	Reduced accumulation of Aβ and lowered oxidative stress	(9)
	PDGFB-APPSwInd transgenic mouse	BHB and ACA subcutaneous injections for 2 months	Reduced amyloid deposition, improved learning and memory and synaptic plasticity	(10)
Epilepsy	adenosine A1 receptors (A1R)-/- and Adk-Tg mice	KD for 3 weeks	Reduced seizure frequency	(11)
	Kcna1-/- mice	KD diet, BHB administration	Reduced spontaneous recurrent seizures	(12)
	6-Hz induced seizure model and Kcna1-/-, mice	KD 2-14 days	Elevated seizure threshold, decreases seizure duration and frequency	(13)
Glaucoma	DBA/2J mutant mouse with elevated intraocular pressure	KD for 8 weeks	Increased neuronal survival	(14)
	NMDA-induced RGC death, rat	KD for 3 weeks	Increased RGC survival in juvenile not adults	(15)
	NMDA-induced RGC death, rat	Daily BHB or ACA intraperitoneal injection for 21 days	Increased RGC survival	(16)
	Blast pressure-induced ocular injury, mice	KD beginning 2 weeks prior to injury	Increased neuronal survival, decreased gliosis	(17)
MS	Experimental autoimmune encephalomyelitis (EAE) mouse	KD up to 30 days	Increased spatial learning and memory, improved motor ability and reduced lesion size	(18)
	Cuprizone-induced demyelination mouse	KD up to 35 days	Improved motor and behavioral deficits, increased mature oligodendrocytes and reduced hippocampal demyelination	(19)
Parkinson's Disease	MPTP mouse	BHB infusions	Decreased dopaminergic neurons degeneration and reduced motor deficits	(20)
	MPTP mouse	KD prior to injury	Improved motor function	(21)
	6-OHDA rat	KD for 7 weeks	None	(2)
	6-OHDA rat	KD for 2 weeks prior to and after injury	Increased neurons	(22)
Retinal degeneration	rd10 model of retinitis pigmentosa, mouse	KD+low protein 1 week prior to disease onset	Increased retina function and thickness	(23)
	Pde6a D670G model of retinitis pigmentosa, mouse	KD for 1 week	Improved retinal histology	(24)
SCI	C5 spinal hemi-contusion, rats	KD initiated 2 weeks prior to injury	Motor recovery, reduced lesion size	(25)
Stroke	Endothelin-1 model of induced ischemia, rat	KD prior to injury	Improved mobility	(26)
	Cardiac arrest-induced cerebral ischemia, rat	KD for 25 days prior to injury	Prevented neurodegeneration	(27)
	Middle cerebral artery occlusion, rat	Intravenous BHB administration after injury	Reduced cerebral infarct area and lower neurological deficits	(28)

**Table 2:** Summary of key published clinical studies examining the beneficial effects of the KD in neurological diseases.

**Disease**	**Trial type**	**KD duration**	**Outcome**	**References**
Alzheimer's disease	Randomized comparison of KD to high carbohydrate diet	6 weeks	Improved verbal memory performance	(29)
	Single-arm	12 weeks	Improved cognitive scores	(30)
	Randomized crossover	12 weeks	Improved quality of life, no improved memory	(31)
Dravet syndrome	Prospective study	3-12 months	Reduced seizures and hyperactivity	(32)
Epilepsy	Randomized, prospective and controlled	3 months	Reduced seizure frequency	(11)
Pharmacoresistant epileptic encephalopathy	Prospective study	12 weeks	Improved cognitive function, reduced seizure frequency	(33)
MS	Single-arm	6 months	Significantly improved fatigue and depression scores	(34)
	Randomized three-armed parallel grouped, single center, controlled	6 months	Improved quality of life and overall health	(35)
Parkinson's disease	Randomized, controlled, parallel-group trial	8 weeks	Small increase in cognitive abilities but no change in motor function	(36)

### Epilepsy

Neal et al. (11) reported the first randomized, prospective and controlled clinical trial for treatment-intractable childhood epilepsy in which 3 month administration of a KD resulted in significant reduction in seizure frequency. The KD was also shown in smaller studies to reduce onset and frequency of seizures in other childhood seizure syndromes such as Dravet syndrome, myoclonic-atonic epilepsy and other conditions (1). The use of rodent models of epilepsy confirmed that the KD significantly reduces seizures and provided clues to potential mechanisms contributing to decreased neuronal excitability. For example, Masino et al. (37), using a transgenic mouse model with spontaneous seizures, found that 3-week administration of the KD reduced seizure frequency in a process that required activation of adenosine A1 receptors (A_1_Rs). The anti-epileptic effect of the KD was lost in A_1_R knockout mice and in mice that were treated with an A_1_R inhibitor (37). In another epilepsy mouse model, the Kcna1-null mouse, seizure reduction was promoted by direct administration of BHB and depended on alterations in mitochondrial permeability transition (12). Furthermore, Olson et al. (13) used two mouse models of epilepsy to identify specific gut microbial species that are influenced by the KD and contribute to seizure reduction by modifying host production of neurotransmitters. Although the beneficial effect of the KD in adults and additional epilepsy subtypes are not yet established by clinical trials, ClinicalTrials.gov lists nearly 40 prospective randomized trials currently recruiting or completed that are investigating effectiveness and safety of KDs in treating epilepsy. Therefore, more information on the benefits of the KD for epilepsy will likely be reported in the future.

### Multiple Sclerosis

Several ongoing randomized controlled studies are listed on Clinicaltrials.gov that are testing safety, tolerability and effectiveness of the KD as a therapeutic intervention for MS. Reports from several pilot studies suggest the KD may benefit patients with MS. For example, Brenton et al. (34) demonstrated that patients on a modified KD for 6 months had no worsening symptoms and showed significant improvements in both fatigue and depression scores. Additionally, evidence for patients with relapsing-remitting MS from a three-armed parallel grouped, single center, controlled and randomized clinical pilot trial showed that being on the KD for 6 months led to improvements in quality of life and overall health (35).

Additional support for a neuroprotective effect of the KD comes from two mouse models of MS. Kim et al. (18) used the experimental autoimmune encephalomyelitis (EAE) mouse model fed a KD with a high fat to carbohydrate plus protein ratio of 6.3:1, which led to increased spatial learning and memory, improved motor ability and reduced lesion size compared with EAE mice fed a standard diet. These improvements were associated with reduced pro-inflammatory responses in brain tissue, including lower cytokine and chemokine levels and lower macrophage and microglia numbers, as well as decreased formation of reactive oxygen species (ROS) (18). Neuroprotection was also observed in the cuprizone-induced demyelination mouse model of MS using mice on a more tolerable fat to carbohydrate plus protein ratio of 3:1 for 5 weeks (19). Animals on the KD had significantly improved motor and behavioral deficits, increased numbers of mature oligodendrocytes and reduced hippocampal demyelination. Elevated expression of myelin basic protein and other myelin associated markers were observed, suggesting that the KD protected against demyelinating pathology (19). Although extrapolations from animal models to human MS patients must be made cautiously, these pre-clinical studies suggest that the KD may potentially reduce MS pathology and ameliorate associated symptoms.

### Alzheimer's Disease

Clinicaltrials.gov lists a dozen ongoing or completed clinical trials that investigate the effectiveness of classic and modified KDs on reducing cognitive impairment. Several published reports have indicated mild improvements in cognitive function, with the ApoE4 genotype influencing the extent of improvement [see (38) for a comprehensive review]. For example, Krikorian et al. (29) demonstrated in a small group of patients that 6 weeks on a KD improved verbal memory performance compared with patients on a high carbohydrate diet. Similarly, a single-arm study of patients on the KD for 12 weeks showed improved cognitive scores, although comparison to a control group was lacking (30). A recent randomized crossover trial showed that 12 weeks on a modified KD led to significantly improved daily function and quality of life, although not significant improvements in memory (31). These results are promising, particularly if the benefits are sustained in longer term studies.

The effectiveness of the KD has been analyzed in several animal models of Alzheimer's disease (AD) but the beneficial effects were shown to vary widely. Administration of BHB in the 3xTgAD mouse model enhanced energy use in the hippocampus and reduced oxidized proteins and lipids, suggesting correction of metabolic defects associated with AD (7). Furthermore, APP/PS1 and Tg4510 mice on a KD for 3 months showed improved locomotor activity, although no improvement in learning or changes in amyloid or tau deposits were detected (8). In comparison, a study using 7 week oral administration of the glucose analog 2-deoxy-D-glucose, which blocks glycolysis and induces ketosis, in the 3xTgAD mouse model led to reduced accumulation of Aβ and lowered lipid peroxidation and oxidative stress response proteins, but did not decrease tau hyperphosphorylation (9). Furthermore, Yin et al. (10) used daily administration of BHB or ACA for 2 months in a mutant APP mouse model and demonstrated lower oxidative damage and reduced β-amyloid deposition, which was associated with improved learning and memory and synaptic plasticity. Therefore, the effectiveness of the KD appears to be inconsistent among different animal models of AD and may be influenced by the method of inducing ketosis. Additional studies to clarify the effect of the KD and ketone bodies on AD pathology are needed.

### Parkinson's Disease

Several pilot clinical studies have reported findings on the effect of nutritional ketosis on reducing PD symptoms. For example, consumption of a KD for up to 8 weeks resulted in small increases in cognitive abilities but not motor functions compared with a low-fat diet (36). However, whether the KD improves symptoms in animal models of PD is controversial. In an early study with the commonly used MPTP neurotoxicity mouse model, Tieu et al. (20) demonstrated that BHB infusions resulted in a small but significant rescue of dopaminergic neuron degeneration and reduced motor deficits. Another study using the MPTP model indicated that mice fed the KD also showed improved motor function and lower inflammation (21). In contrast, a rat model of induced PD-like lesions from 6-OHDA injection into the medial forebrain bundle showed no protection of dopaminergic neurons or functional improvement after 7 weeks on the KD despite increased brain BHB levels (2). Although increased number of tyrosine hydroxylase-positive neurons were reported in a different study using 6-OHDA injected rats fed the KD, neuronal apoptosis or motor function were not directly measured (22). Therefore, the literature does not yet support a neuroprotective effect of the KD in PD but it may indirectly improve motor performance. Because motor improvement was not demonstrated in the human pilot studies, longer studies may be necessary, and the possibility remains that these improvements may not translate from animals to humans.

### Spinal Cord Injury

To date, there have been no completed clinical trials reported in the USA or European Union although promising results of the KD have been obtained in animal models of SCI. In a study on rats after C5 spinal hemi-contusion, Streijger et al. (25) found that administration of the KD promoted motor recovery, measured by ipsilateral forelimb use and range of motion, and was associated with smaller lesion sizes compared to rats fed the control diet. Blocking MCT activity prevented neuroprotection by the KD. Notably, improved forelimb function was maintained after the animals were switched back to a standard diet. This result provides compelling evidence to investigate whether the KD leads to functional improvements in patients after SCI.

### Stroke

Several clinical trials investigating possible benefits of the KD have been completed according to Clinicaltrials.gov but conclusions have not yet been reported. No clinical trials are registered in the EU. Animal studies using various stroke models have demonstrated reduced pathology due to a KD or direct administration of exogenous BHB prior to injury. Shaafi et al. (26) used the endothelin-1 model of induced ischemic stroke to demonstrate that rats fed the KD shortly before stroke induction had improved mobility, indicating a benefit of diet preconditioning. Similarly, rats fed the KD for 25 days prior to injury were protected from neurodegeneration caused by cardiac arrest-induced cerebral ischemia (27), and intravenous administration of BHB in rats after the initiation of middle cerebral artery occlusion significantly reduced cerebral infarct area and neurological deficits (28). However, it is still unknown whether providing the KD after stroke injury would be protective and whether the mechanisms of neuroprotection differ between providing the KD pre-injury or post-injury.

### Glaucoma

There are no clinical trials listed at clinicaltrials.gov (USA) or clinicaltrialsregister.eu/ (European Union) on the use of a KD to treat glaucoma. Neuroprotective effects of the KD have been shown in mouse and rat models of glaucoma. In a study using the DBA/2J mouse model of elevated intraocular pressure and secondary glaucoma, mice fed the KD for 8 weeks showed significantly increased retinal ganglion cell (RGC) survival and axon number compared to mice fed the control diet (14). This neuroprotection was associated with increased mitochondria and reduced expression of pro-inflammatory molecules in the retina and optic nerves. In a second model, the NMDA-induced RGC death model, pretreatment of juvenile but not adult rats with a KD for 21 days led to increased RGC survival (15). An age-dependent neuroprotective effect of the KD was also observed in another rat CNS injury model, cortical impact injury, and may be related to variations in MCT expression and ketone uptake into the brain (39). Another study using the NMDA toxicity model showed that intraperitoneal injections of BHB and acetoacetate in rats also reduced RGC degeneration although age was not investigated as a variable (16). Additionally, mice fed the KD prior to or after pressure-induced ocular injury had decreased optic nerve gliosis, reduced optic nerve degeneration and higher visual evoked potential amplitudes (17). Together, these studies provide support for investigating the KD as a potential therapy for glaucoma.

### Retinal Degenerations

There are no US or European clinical trials on the effect of the KD on common retinal degenerations, such as age-related macular degeneration or retinitis pigmentosa. However, investigation in mouse models of retinal degenerations revealed neuroprotection by the KD. Ryals et al. (23) examined the effect of a ketogenic combined with protein restriction using the rd10 mouse model of autosomal recessive retinitis pigmentosa, which causes early onset and severe photoreceptor degeneration. The combination of a protein-restricted KD led to high BHB levels, increased retinal function and increased photoreceptor layer thickness. This protective effect was observed after the mice were placed on the diet 1 week before the onset of photoreceptor degeneration, indicating rapid induction of the neuroprotective response. In contrast, rd10 mice on a KD with typical protein levels did not show neuroprotection, while wild-type mice fed the protein-restricted KD showed reduced photoreceptor function with no corresponding change in photoreceptor survival. The authors proposed that the low protein KD protected the retina by reducing phototransduction, lowering calcium influx and decreasing subsequent ROS production (23). A second study using a similar mouse model of autosomal recessive retinitis pigmentosa (Pde6a D670G mutation) also demonstrated increased photoreceptor survival after 1 week on the standard KD, measured by improved retinal histology, although improved photoreceptor function was not found (24). Therefore, these studies indicate that even a short time on the KD can lead to measurable benefits to the retina in mouse models of inherited retinal degenerations.

## Neuroprotective Mechanisms of the KD

Various molecular mechanisms have been associated with neuroprotective effects of the KD and ketone body administration. Most studies report correlations between cellular pathways and neuroprotection, such as changes in oxidative stress or inflammatory proteins, but whether these pathways are altered as primary or secondary effects of the diet is not determined in many studies and remains controversial. Recently, several elegant studies using inhibitors and mouse knockout strains have provided evidence for direct contributions of specific cellular pathways in mediating neuroprotective effects of the KD. These studies are summarized below.

### Energy Supply Restoration

Neuronal injury often leads to changes in glucose metabolism. The simplest mechanism for neuroprotection by the KD is that ketone bodies serve as alternative fuels for brain metabolism, which maintains mitochondrial function, ATP production and neuronal survival. For example, in the MPTP neurotoxicity mouse model discussed above, BHB induced neuroprotection by preventing the decline in mitochondrial respiration by acting on complex II (succinate-ubiquinone oxidoreductase) and restoring ATP production (20). The ketogenic diet also promoted complex II and IV activities in a glaucoma mouse model, which increased energy production and promoted neuronal survival (40). Increased mitochondrial respiration induced by BHB administration was associated with higher ROS from oxidative phosphorylation in the MPTP model, although ROS levels were not elevated in the KD fed glaucoma model, suggesting additional benefits from the diet counter elevated ROS. Therefore, the production of ketones from the KD can help cells avoid energy deficits after neuronal injury and prevent the cascade of events that lead to neuronal death.

AMP-activated protein kinase (AMPK) senses cellular energy levels and is activated when energy levels are low, which serves to reduce ATP consumption and promote ATP production. AMPK regulates inflammation by activating the major inflammatory regulator NF-κB, which leads to transcription of pro-inflammatory proteins, including TNFα, IL-1β and IL-6 (41). Analysis of retinas from a glaucoma mouse model fed the KD demonstrated reduced AMPK activation, which was associated with lower NF-κB p65 nuclear translocation and decreased expression of pro-inflammatory molecules (40). The authors concluded that the anti-inflammatory effect of the KD in the glaucomatous retina is dependent on its ability to restore normal ATP production through the supply of ketone bodies and consequential changes in AMPK activation.

### Induction of Anti-inflammatory Pathways

**Figure 2:**
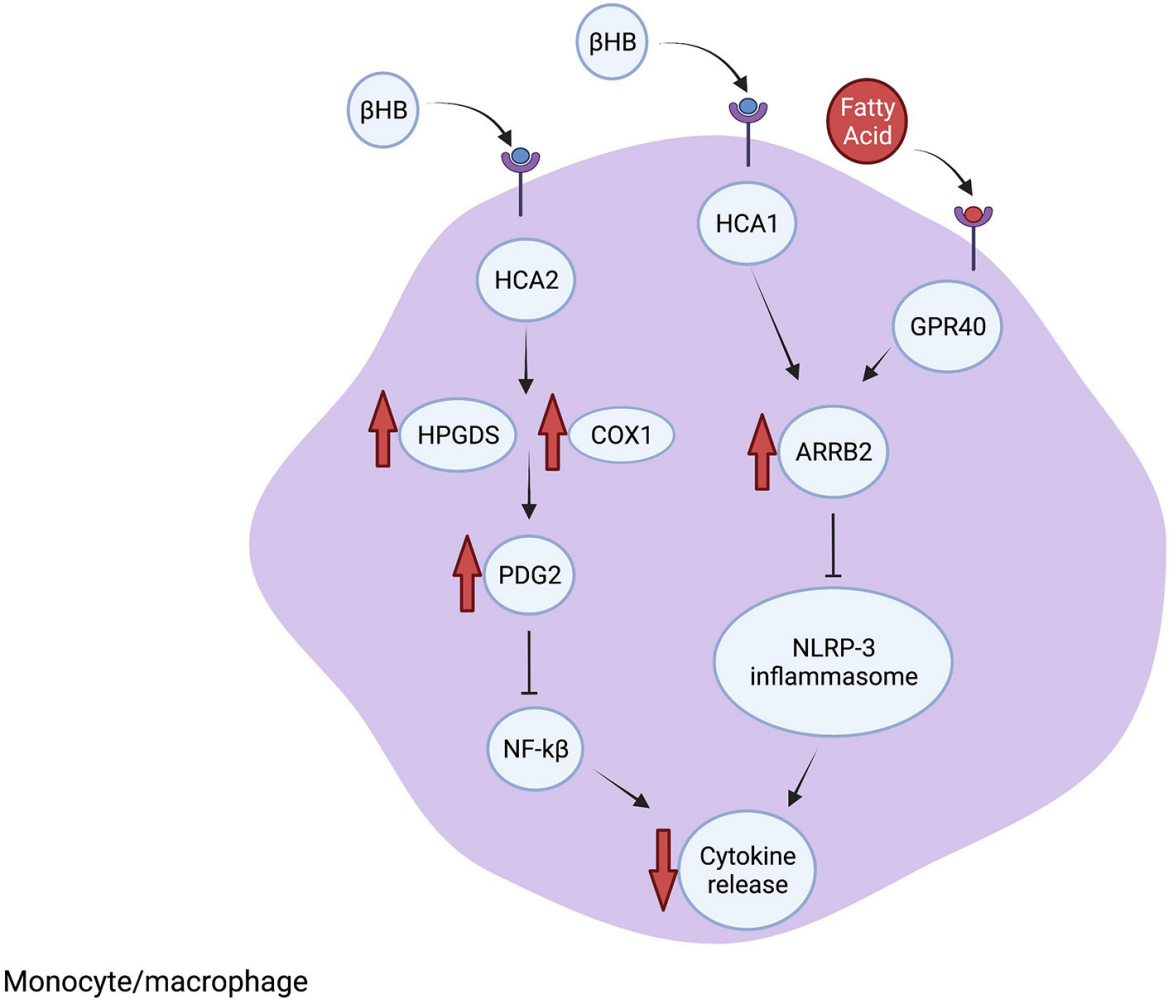
The KD reduces inflammation. The scaffolding protein ARRB1 is elevated after BHB binds to HCA1 or fatty acids (FA) bind to GPR40. Increased ARRB1 inhibits the NRLP-3 inflammasome formation, which reduces cytokine processing and release. Furthermore, BHB interacting with HCA2 leads to increased PDG2 synthesis through increased HPGDS and COX1, which leads to inhibition of NF-kB and suppressed inflammatory signaling. Additional anti-inflammatory mechanisms are described in the text.

Neuroinflammation is an intrinsic response to neuronal injury and disease. Inflammatory cells, including microglia, macrophages, astrocytes and other non-neuronal cells, function together to repair neuronal damage by enhancing phagocytosis and secreting neuroprotective and anti-inflammatory molecules. However, unregulated neuroinflammation can cause further neuronal loss from elevated ROS levels and release of neurotoxic and pro-inflammatory cytokines. The neuroprotective effects of the KD in animal models are often correlated with reduced pro-inflammatory signaling and are characterized by lower microglia numbers, decreased expression of the inflammation inducer NF-κB p65 protein and reduced levels of pro-inflammatory molecules such as TNFα. Until recently, it was unknown whether these inflammatory changes are directly induced by ketones or secondary to other processes. In the studies described below, pharmacologic and genetic knock-outs of specific molecular pathways were used to demonstrate the importance of several mediators of inflammation that are altered directly by ketone bodies, including the receptors HCA1 and HCA2, GPR40 fatty acid receptor and adaptor protein ARRB2 (Figure 2). Further, BHB itself functions as a signaling molecule in addition to serving as an energy source. Therefore, evidence is accumulating that the KD, and more specifically BHB and its metabolites, directly reduce pro-inflammatory responses leading to neuroprotection.

Inflammatory signaling in the CNS is often mediated by the NLRP3 inflammasome. Upon activation by microbial PAMPs and other stress-related molecules, NLRP3 leads to the activation of caspase-1 followed by cleavage of pro-IL-1β into its active IL-β form, which promotes further inflammatory signaling. The NLRP3 inflammasome also interacts with NF-κB and induces transcription of pro-inflammatory cytokines which promotes further activation of NLRP3 (42). Investigations in cultured macrophages, human monocytes and mouse models of inflammatory diseases demonstrated that BHB inhibits NRLP3 inflammasome activation through a mechanism that involves blocking potassium efflux and preventing ATP-induced ASC oligomerization, which are both needed for inflammasome assembly (43). The other ketone bodies, acetoacetate, acetate and butyrate, did not inhibit inflammasome activity. The authors concluded that metabolites such as BHB may suppress ATP-mediated inflammatory responses, particularly involving NRLP3, during glucose-depleted states as an intrinsic mechanism to reserve ATP for the activity of ketone-dependent organs such as the brain and heart (43).

BHB binds directly to hydroxycarboxylic acid receptor 2 (HCA2) (also called GPR109A) on adipocytes (44). HCA2 is also expressed on neutrophils, macrophages and other cells in the brain, expanding the potential target cells influenced by BHB and the KD. In the MCAO mouse model of ischemic stroke, BHB was shown to activate HCA2 in macrophages that infiltrate the brain after injury and the protective effect of the KD and BHB was lost in HCA2 knock-out mice. Mechanistically, BHB activation of HCA2 resulted in activation of the enzymes HPGDS and COX1 which induces prostaglandin D2 (PDG2) synthesis. PDG2 and its conversion product 15d-PGJ2 are neuroprotective by reducing NF-κB activation, decreasing proinflammatory genes and by activating the transcription factor PPARgamma (45).

Lin et al. (46) identified mechanisms downstream of the fatty acid receptor GPR40 that inhibit proinflammatory pathways using a rat traumatic brain injury model treated with omega-3 fatty acids, which are a common component of the KD. Inhibiting GPR40 using chemical antagonists prevented the anti-inflammatory effect of the fatty acid-enriched diet. The GPR40 receptor interacts with the scaffolding protein ARRB2, which also interacts with and directly inhibits NLRP3 activity. Treatment with omega-3 fatty acids resulted in ARRB2-mediated inhibition of NLRP3, reducing IL-1β inflammatory signaling and promoting neuronal survival. Furthermore, reducing ARRB2 expression or knocking out NLRP3 inhibited ARRB2-NLRP3 binding and prevented the anti-inflammatory effect of fatty acids. Therefore, GPR40-ARRB2-inflammasome inhibition is likely an important mediator of the neuroprotective effect of dietary fats. Although the KD differs from the omega-3 enriched diet, and directly comparing the diets cannot be done, the findings of Lin et al. raise the possibility that specific fatty acid components of the KD may contribute to the observed anti-inflammatory effects. Additionally, in a mouse model of glaucoma, the KD was associated with increased expression of ARRB2 and its interacting protein and BHB target HCA1, which led to reduced levels of NLRP3 and IL-1β, consistent with inhibited NLRP3 inflammasome function (40). Therefore, the KD reduces inflammation through binding of BHB-HCA1 and fatty acids-GPR40, which leads to ARRB2-dependent suppression of NRLP3 inflammasome function (Figure 2).

The KD and BHB were also demonstrated to have direct effects on microglia ramifications, causing microglia to polarize toward the M2-like neuro-reparative and protective phenotype (40). BHB was shown to mediate this change in microglia morphology by stimulating Akt signaling, which involved inhibition of HDACs but not HCAR2 (47). Consequently, BHB may promote anti-inflammatory signaling by altering microglial morphology. The KD was also shown to increase anti-inflammatory responses in glaucomatous mice, evidenced by increased expression of Arginase 1, a marker of neuroprotective anti-inflammatory microglia, as well as increased expression of the anti-inflammatory cytokine IL-4 in retinas and optic nerves (40).

Cellular mechanisms induced in low energy states also regulate inflammatory responses (48). Energy metabolism influences inflammation through changes in the cytosolic NADH:NAD+ ratio (48). Shen et al. used the glycolytic inhibitor 2DG to mimic changes in glycolytic flux from the KD and demonstrated that reduced glucose usage leads to lower NADH:NAD+ ratio, which inhibits the NAD(H) sensitive transcriptional co-repressor CtBP in microglia. Reduced CtBP activation has an anti-inflammatory effect through decreased activity of p300 and reduced NF-κB binding to the promoters of proinflammatory genes (49). Therefore, the KD may alter inflammation through bioenergetic changes in the NADH:NAD+ ratio.

Complicating the effect of the KD on inflammation is the finding that acetoacetate activates inflammatory pathways through TNFα, which is in contrast to the anti-inflammatory properties of BHB (50). Additionally, high concentrations of BHB induce inflammatory signaling molecules NF-κB, TNFα, IL-6 and IL-1β (51). Therefore, the overall effect of the KD on inflammation is influenced by local concentrations of ketone bodies in the affected tissues and the ratio of BHB to acetoacetate.

### Reducing Oxidative Stress

Oxidative stress is a secondary effect of neuronal injury and is caused by mitochondrial dysfunction and subsequent production of reactive oxygen species (ROS), reactive nitrogen species (RNS) and reactive electrophile species (RES), all of which have been associated with neurotoxicity and neuronal death in neurodegenerative diseases (52). Blocking oxidative stress using chemical or genetic modulators reduces excitotoxicity and mitochondrial dysfunction, leading to cellular protection in animal models of many neurodegenerative diseases (52).

The KD is associated with decreased markers of oxidative stress in animal models, including reduced nitric oxide synthase-2 (NOS2) in the retina of glaucomatous mice (40), reduced retinal superoxide and elevated anti-oxidant proteins such as SOD2 in the retina after blast injury (17) and increased antioxidant responses and regulation of ROS in rat cortex after impact injury and kidney tissue from mice treated with BHB (53, 54). Physiological concentrations of BHB are able to scavenge ROS and hydroxyl radicals and directly reduce cellular ROS levels, which preserves mitochondrial functioning and increases cell survival (55). For example, in the cuprizone-induced demyelination mouse model of MS, the KD led to increased activity of glutathione peroxidase, an enzyme that breaks down H_2_O_2_, and reduced expression of the oxidative stress marker malondialdehyde; these molecular changes in oxidative stress markers were correlated with improved myelination in the hippocampus (19). Interestingly, brain region-specific changes in antioxidant enzyme activities were noted in rats fed a KD, with the hippocampus showing increased levels of antioxidant enzymes that were not observed in the cerebral cortex or cerebellum (56). However, the implication for neuroprotection in specific brain regions is not known.

**Figure 3:**
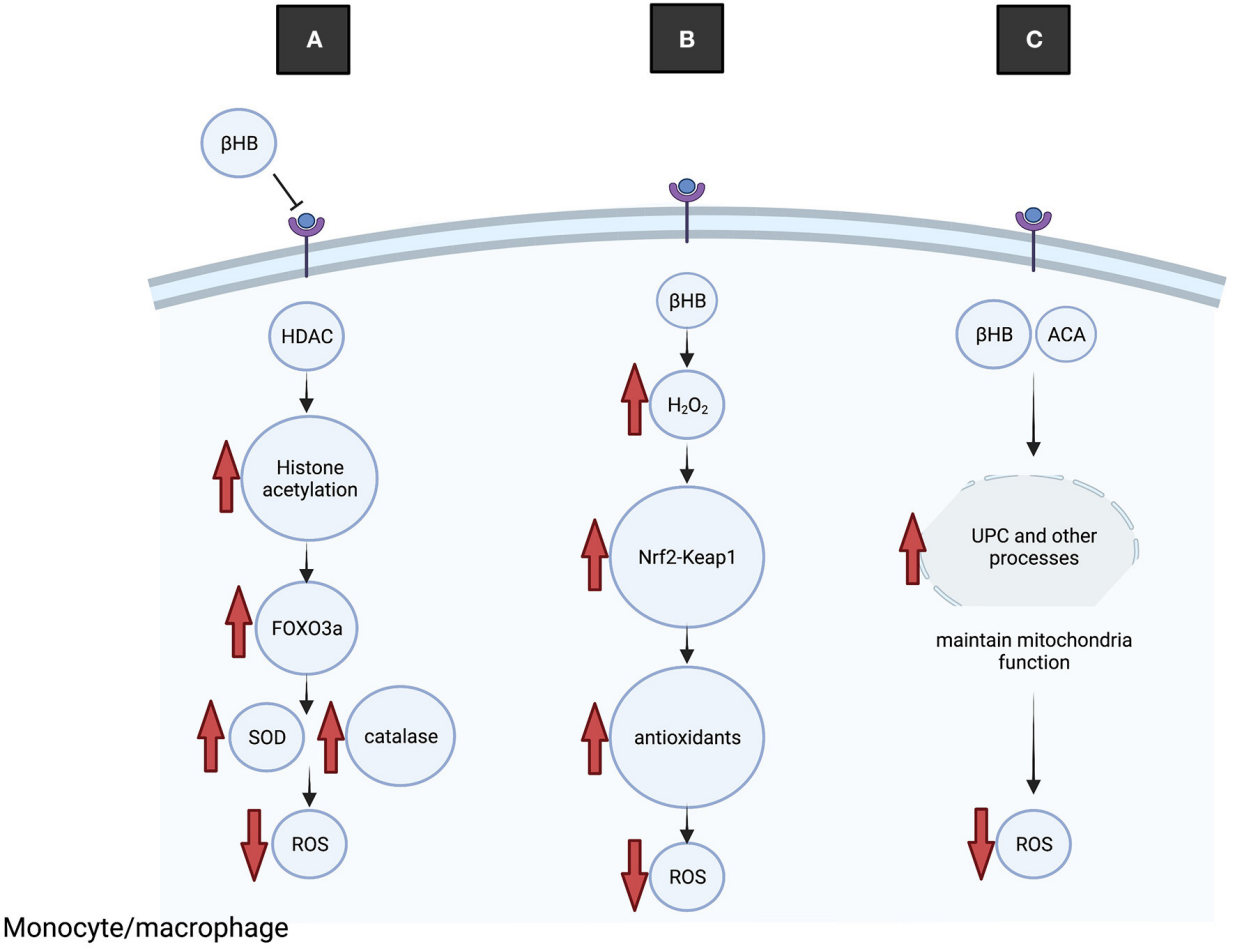
The KD reduces oxidative stress. **(A)** BHB binds to and inhibits HDAC, leading to increased histone acetylation at the promotor of the transcription factor Foxo3a and increased expression. Foxo3a induces expression of antioxidant proteins SOD and catalase, leading to reduced ROS. **(B)** Transiently elevated H_2_O_2_ leads to dissociation of Keap1 from Nrf2, nuclear translocation of Nrf2 and transcription of antioxidant genes, leading to reduced ROS. **(C)** Ketone bodies increase UCP activity, which improves mitochondrial functioning and reduces production of ROS.

Several molecular mechanisms that contribute to KD-mediated reduction of oxidative stress have been described (Figure 3). Mice fed the KD had higher mitochondrial respiration rates and increased expression of mitochondrial uncoupling proteins (UCP) than mice on a control diet (57). Increased UCP activity reduces mitochondrial membrane potential and lowers production of ROS and reactive oxygen nitrogen species. Ketone bodies may increase UCP expression through regulating Sirtuin 1 activity (58), potentially via altered NAD+ levels (59), although the precise mechanisms are not yet known.

Nrf2 is a key endogenous protective transcription factor that leads to increased transcription of antioxidative enzymes including glutathione reductase, heme oxygenase-1, thioredoxin and peroxiredoxin. An interesting study on rats fed the KD demonstrated acute and transient increases in oxidative (H_2_O_2_) and electrophilic (lipid peroxidation end product 4-HNE) stress in the brain, which appears to conflict with the general antioxidant properties of ketone bodies (60). However, the elevated H_2_O_2_ levels were shown to stimulate the Nrf2 antioxidant pathway through Nrf2-Keap 1 redox sensing, and elevated Nrf2 was detected in brain and liver tissue after 1 week on the diet. Because H_2_O_2_ increases Nrf2 binding to DNA, a potential neuroprotective mechanism is that transient elevation of H_2_O_2_ and altered redox states from the KD serve to activate Nrf2 leading to induction of antioxidation pathways (60).

### Gut Microbiome Alterations

The gut microbiome has been associated with several neurological disorders, such as Alzheimer's disease, epilepsy, Parkinson's disease and multiple sclerosis. Changes in the gut microbiome are observed in children on the KD to prevent seizures as early as 1 week into the diet (61), although the significance to improved epilepsy symptoms is not understood. Additionally, the colonic microbiome of MS patients on the KD for 6 months showed increased bacterial concentrations and bacterial species diversity (62). Microbial species in the gut secrete various metabolites, such as tryptophan, short-chain fatty acids, neurotransmitters and immunomodulatory molecules. Therefore, changes in the microbiome caused by the KD may also affect microbial-derived molecules that play a role in CNS homeostasis and possible neuroprotection (63).

Olson et al. (13) used two epilepsy mouse models fed a KD to determine the role of specific gut microbial species influenced by the diet. Mice fed the KD had significantly fewer seizures than mice fed a control diet, as well as increased levels of the gut bacteria species *Akkermansia* and *Parabacteroides*. These bacteria caused reduced gamma-glutamyltranspeptidase in the stomach and lowered gamma-glutamylated amino acids in the colon and vascular system, which led to higher GABA production in the brain and an elevated seizure threshold (13). The authors treated the mice with antibiotics to deplete the gut microbiota or reared them in germ-free conditions which confirmed that changes in the microbiome were responsible for the ketogenic anti-epileptic effect. Additionally, transplanting samples of microbiota from mice fed the KD into control mice, as well as transplanting *Akkermansia* and *Parabacteroides* bacteria into mice fed the control diet, resulted in similar levels of seizure protection as mice directly fed the KD (13). Therefore, the anti-convulsant properties of the KD in these mice were mediated by changes in specific bacterial species in the gut. In humans, the gut microbiome is affected by many different factors, making the connection between KD, gut microbes and neuroprotection currently only correlative and requiring further study.

### Epigenetic Mechanisms

BHB regulates the epigenome by modifying histone acetylation by inhibiting HDAC or activating Sirtuin 1. BHB increases histone acetylation by inhibiting class I histone deacetylases (HDACs), which are a family of proteins that regulate transcription of numerous genes through histone modifications. Removal of acetyl groups by HDACs alters DNA conformation and leads to transcriptional repression. Mice treated directly with BHB or placed on caloric restriction to elevate endogenous BHB showed evidence of HDAC inhibition and increased histone acetylation of promoters of oxidative stress resistance genes, including the transcription factor Foxo3a and metallothionein 2 (54). Targets of Foxo3a include the antioxidant genes mitochondrial superoxide dismutase and catalase, which were also elevated in BHB-injected mice. However, these studies were performed in kidney tissue and it remains to be determined whether BHB also inhibits HDAC in the CNS, and whether induction of Foxo3a-related antioxidants after HDAC inhibition contributes to neuroprotection by the KD (54). In another study, analysis of liver cells in mice fed the KD showed induction of Sirtuin 1 activity (64), an NAD+-dependent protein deacetylase. Therefore, the role of acetylation-dependent transcription regulation in mediating neuroprotection by the KD may be influenced by opposite effects of ketones on HDAC and Sirtuin 1.

BHB itself directly modifies lysine residues on histones in regions of active gene promoters (65) in a process called β-hydroxybutyrylation. A recent study demonstrated that the acyltransferase p300 adds β-hydroxybutyrate to lysine, while HDAC1 and HDAC2 remove it (66). Over 1,300 other proteins in addition to histones were identified as targets of ketone-related modifications, suggesting that this is a major regulatory pathway that is potentially altered by the KD (66).

## Discussion

### Controversies and Adverse Effects of the KD

The mechanisms of neuroprotection described above have often been studied in a single animal model and the generalizability to other sources of neuronal injury and different neuronal tissues are unknown. Notably, the effect of ketone bodies on antioxidants has been shown to differ across brain regions (56) and exhibit age-specific effects (15), which may underly variations in outcomes of the KD among different studies. As noted above, high concentrations of BHB were found to induce pro-inflammatory signaling which is the opposite effect of lower concentrations (51); because of this dose-dependent BHB effect on inflammation, it is important to measure serum and/or tissue BHB levels to properly assess the effect of the KD. Additionally, beneficial effects of the KD in animals have been shown to not always translate to a benefit in humans, for example, motor improvements were shown in rat models of PD but not patients with PD. Therefore, extrapolating the results of studies from animals to humans must always been done cautiously. Furthermore, it is important to consider that molecular mechanisms that lead to reduced inflammation and lower oxidative stress may change with longer durations of the diet, and the reported short-term mechanisms of neuroprotection may not necessarily contribute to its long-term effects.

Adopting the KD would appear to contradict current dietary guidelines that recommend reduced intake of total fat. However, potential negative effects of elevated dietary fat intake appear to be balanced by the beneficial effects of reduced carbohydrates and elevated ketones. The KD is popular with the general public for weight loss and side effects generally are minor. However, long-term strict adherence to the diet is associated with adverse effects, and gastrointestinal issues (such as diarrhea, abdominal distention, reflux), cardiac problems (arrhythmia) and poor growth have been noted in pediatric epilepsy patients on the KD (67–69), although a confounding variable is that these patients often have underlying metabolic and other health issues. Less common but more severe side effects include specific nutrient deficiencies, kidney stones, bone fractures and increased infections, which can be treated with standard therapies and appropriate vitamin and nutrient supplementation while maintaining the diet (70). For example, several case reports indicate that complications of the KD included protein–losing enteropathy, edema and hypoalbuminemia; in a recent case report these issues were resolved by reducing the fat to carbohydrate ratio in the diet (71). Another report demonstrated that most gastrointestinal and nutrient deficiencies occurring early (< 4 weeks on the diet) and complications such as anemia and cardiomyopathy occurring late (> 4 weeks) were transient and successfully managed (69).

The effect of the KD on growth and skeletal health was reviewed by Merlotti et al. (72). Multiple studies in rats on the KD and modified KD combined with other treatments indicated reduced biomechanical function, reduced bone mineral density and increased osteoporosis. In contrast, most studies with patients on the KD showed no or minimal negative effects on growth, bone mass or molecular markers of bone health over 24 months (72). In a long-term retrospective study of pediatric patients on the diet for 8 years, a low but significant frequency of individuals with reduced bone mineral density was observed (73). This observation may be explained by a potential relationship between the genetic cause of epilepsy and skeletal damage, for example GLUT1 gene mutations (72). Additionally, in a study on children who had followed the KD for a median of 6 years and then discontinued the diet at least 6 months prior to the study, no long-term effects were observed on physical growth, lipid levels and other measurements (74). Therefore, current evidence does not indicate significant effects on bone metabolism, although most studies may not be long enough duration to determine increased risk of fractures or altered bone health.

## Future Research Directions

There are several key gaps in knowledge about the KD that require further investigation. First, determining whether the KD reduces pathology and alleviates neurological symptoms of the diseases discussed above should be clarified with randomized clinical trials using blinded clinical assessments whenever possible and appropriate control diets. Second, due to potential side effects and potential non-compliance with the diet, it is important to consider whether exogenous ketones can be used to achieve therapeutic levels of circulating ketone bodies, as suggested from studies in animal models of disease. Third, multiple cell types react to and metabolize ketone bodies, and further investigation into whether inflammatory cells, glia and neurons respond differently to ketone bodies should be explored. Fourth, understanding mechanisms of brain region-specific and age-dependent neuroprotection of the KD may reveal new insights into the regulation of neuronal survival pathways and the influence of bioenergetic pathways. Fifth, identification of potential mechanisms of neuroprotection may be used to develop biomarkers or indicators of the effectiveness of the diet, such as changes in specific plasma cytokines. Sixth, further detailed characterization of the mechanisms that modify the effect of KD-induced neuroprotection is needed, including the role of expression levels of relevant transcription factors, BHB receptors and MCT transporters, local ketone body concentration, gut microbiome species and mitochondrial density and function, all of which could contribute to variation in the response to KD. Finally, additional studies should determine whether modified KDs, which are less restrictive and may be easier to adopt, would also have beneficial effects for the conditions described above including investigation of improving quality of life in the aging population.

## Conclusions

As described above, the KD has been used for many years to reduce seizures in treatment-intractable pediatric epilepsy and was confirmed to be effective in a randomized control clinical trial (11). Furthermore, recent studies have demonstrated neuroprotection from the KD or direct administration of ketone bodies in numerous animal models of neurological disease, including stroke, glaucoma and spinal cord injury, which raises the possibility that the KD may reduce symptoms in patients with these conditions. However, the existing literature on the effectiveness of the KD in several neurological disorders shows that it reduces symptoms and improves quality of life in several diseases when tested in small pilot studies, such as MS and Alzheimer's disease, but results from larger randomized clinical trials have not yet been reported. Furthermore, the duration of the beneficial effects is unknown because all studies have been short-term, except for analysis of side effects in epilepsy patients. Finally, variations in the composition of the diet, duration of treatments, and frequently small sample sizes in the study groups, make it difficult to compare and draw conclusions across studies. Therefore, although adverse effects of the KD are minimal, evidence does not yet indicate adopting the KD for reducing neurodegeneration in common neurological diseases.

## Author Contributions

The manuscript was conceived and developed by AH. SG, AC, KO, and AH performed the literature review, manuscript writing and editing. Figures preparation were performed by AH and KO. All authors read and approved the final manuscript.

## Funding

Financial support for this study was from NEI R01 EY026546. Institutional support to BPEI was from a Research to Prevent Blindness Unrestricted Grant and an NEI Center Core Grant EY014801.

## Conflict of Interest

The authors declare that the research was conducted in the absence of any commercial or financial relationships that could be construed as a potential conflict of interest.

## Publisher's Note

All claims expressed in this article are solely those of the authors and do not necessarily represent those of their affiliated organizations, or those of the publisher, the editors and the reviewers. Any product that may be evaluated in this article, or claim that may be made by its manufacturer, is not guaranteed or endorsed by the publisher.
